# Genome-Wide Profiling of Small RNAs and Degradome Revealed Conserved Regulations of miRNAs on Auxin-Responsive Genes during Fruit Enlargement in Peaches

**DOI:** 10.3390/ijms18122599

**Published:** 2017-12-13

**Authors:** Mengya Shi, Xiao Hu, Yu Wei, Xu Hou, Xue Yuan, Jun Liu, Yueping Liu

**Affiliations:** 1 College of Plant Science and Technology, Beijing University of Agriculture, Beijing 102206, China; shimengya@agri.gov.cn (M.S.); 201530212017@bua.edu.cn (X.H.); 2 National Agro-Tech Extension and Service Center, Beijing 100125, China; 3 National Key Facility for Crop Resources and Genetic Improvement, Institute of Crop Science, Chinese Academy of Agricultural Sciences, Beijing 100081, China; yuuu.weiii@gmail.com; 4 College of Biological Science and Engineering, Beijing University of Agriculture, Beijing 102206, China; 201530123107@bua.edu.cn (X.H.); 201630123115@bua.edu.cn (X.Y.); 5 Beijing Collaborative Innovation Center for Eco-environmental Improvement with Forestry and Fruit Trees, Beijing University of Agriculture, Beijing 102206, China

**Keywords:** auxin, degradome, fruit development, microRNA, peach

## Abstract

Auxin has long been known as a critical phytohormone that regulates fruit development in plants. However, due to the lack of an enlarged ovary wall in the model plants *Arabidopsis* and rice, the molecular regulatory mechanisms of fruit division and enlargement remain unclear. In this study, we performed small RNA sequencing and degradome sequencing analyses to systematically explore post-transcriptional regulation in the mesocarp at the hard core stage following treatment of the peach (*Prunus persica* L.) fruit with the synthetic auxin α-naphthylacetic acid (NAA). Our analyses identified 24 evolutionarily conserved miRNA genes as well as 16 predicted genes. Experimental verification showed that the expression levels of miR398 and miR408b were significantly upregulated after NAA treatment, whereas those of miR156, miR160, miR166, miR167, miR390, miR393, miR482, miR535 and miR2118 were significantly downregulated. Degradome sequencing coupled with miRNA target prediction analyses detected 119 significant cleavage sites on several mRNA targets, including *SQUAMOSA* promoter binding protein–like (SPL), ARF, (NAM, ATAF1/2 and CUC2) NAC, *Arabidopsis thaliana* homeobox protein (ATHB), the homeodomain-leucine zipper transcription factor revoluta(REV), (teosinte-like1, cycloidea and proliferating cell factor1) TCP and auxin signaling F-box protein (AFB) family genes. Our systematic profiling of miRNAs and the degradome in peach fruit suggests the existence of a post-transcriptional regulation network of miRNAs that target auxin pathway genes in fruit development.

## 1. Introduction

Peach trees are one of the most widely planted *Rosaceae* trees owing to their economic and ecological importance. Peaches originated in East Asia and have been cultivated for more than 3000 years [1]. Due to its relatively small genome (265 Mb), short breeding season and abundant cultivars with various phenotypes, peach is now a model *Rosaceae* plant species for studies of plant development, evolution and comparative genomics [2].

MicroRNAs (miRNAs) are endogenous 20–24 nt small noncoding RNAs found in plants, animals and some viruses. In plants, miRNAs bind to their target miRNAs via base pairing with complementary sequences within the mRNA molecules, and drive cleavage of their targets by binding to Argonaute proteins. As a result of this interaction and cleavage, the miRNA targets are silenced at the post-transcriptional level. During the past decades, miRNAs have been shown to play crucial roles in plant growth, development and stress responses. Recent studies have established that regulatory networks exist between miRNAs and plant hormone signaling pathways for multiple developmental processes [3,4,5,6]. Genomic studies uncovered that the expression of some peach miRNAs was preferentially induced in roots, leaves, flowers and/or fruit in response to stress conditions [7,8,9]. To date, 180 mature miRNAs and 214 miRNA precursors have been collected in peach in the miRBase20.0 database [10]. Most of these miRNAs are evolutionarily conserved.

Across the entire life cycle of many plants, auxin is involved in various developmental and physiological processes, including embryogenesis, morphogenesis of the roots and shoots, vascular differentiation and organ formation [11]. Auxin response factor (ARF) is an important transcription factor family that regulates auxin-responsive genes by directly binding to auxin response elements located in the promoter regions of these genes [12,13]. miRNAs have been shown to directly or indirectly regulate several ARFs. For example, in *Arabidopsis*, miR167 controls the expression levels of *ARF6* and *ARF8* to regulate reproductive processes, while miR160-mediated regulation of *ARF16* and *ARF17* can produce morphological changes [14,15]. In addition, miR167 can degrade *IAA-Ala Resistant 3* (*IAR3*) in *Arabidopsis* roots under osmotic stress [16], whereas miR393 regulates the expression of the auxin receptor *TIR1* and three other closely related auxin signaling F-box proteins (*AFB1*, *AFB2* and *AFB3*) [17]. miR390 can indirectly inhibit the expression of *ARF2*, *ARF3* and *ARF4* in *Arabidopsis* by targeting *TAS3*-derived trans-acting short-interfering RNAs (tasiRNAs) [18]. Another miRNA, miR172, was shown to be a key regulator in fruit growth since its encoding gene was activated by (MCM1, agamous, deficiens and serum response factor) MADS-domain protein and ARFs, which link auxin pathways to fruit morphogenesis [19,20]. In the tomato fruit, miR156, miR172, miR393 and their targets were also detected [21]. Together, these studies indicate that miRNAs specifically affect multiple steps of auxin signal transduction processes in plants [22].

Fruit enlargement is one of the most important developmental processes in peach and other fruit plants. Auxin has long been known to play a central role in regulating ovary wall enlargement and pericarp development, and synthetic auxins can enhance fruit growth via stimulating fruit cell enlargement in some species such as peach [23], plum and loquat [24,25]. In this study, we carried out small RNA sequencing and degradome sequencing analyses of developing peach fruit at the hard core stage following exposure to α-naphthylacetic acid (NAA). Our experiments identified a group of conserved and predicted miRNAs that respond to NAA treatment. Using degradome sequencing and miRNA target prediction analyses, we detected 119 miRNA cleavage sites on target mRNAs, including those for *SQUAMOSA* promoter binding protein–like (SPL), ARF, (NAM, ATAF1/2 and CUC2) NAC, *Arabidopsis thaliana* homeobox protein (ATHB), the homeodomain-leucine zipper transcription factor revoluta (REV), (teosinte-like1, cycloidea and proliferating cell factor1) TCP and auxin signaling F-box protein (AFB) genes. We also verified the abundance of 18 miRNAs using fluorescence stem-loop quantitative real-time PCR (stem-loop qRT-PCR) assay. Our study revealed a post-transcriptional regulation network of miRNAs and their targets in auxin signaling pathways during fruit development in peach.

## 2. Results

### 2.1. Genome-Wide Identification of miRNAs by Small RNA Sequencing in Peach

**Figure 1:**
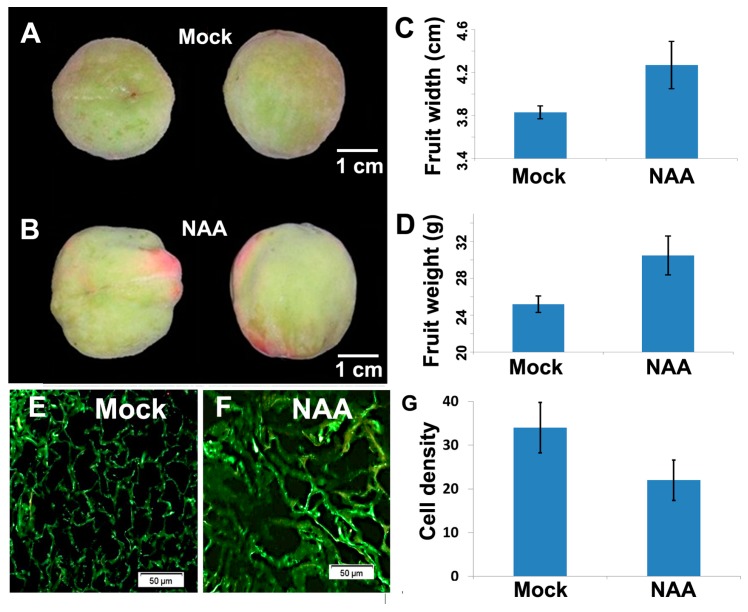
Auxin regulates fruit enlargement in peaches. The development of the peach fruit following a 3-day mock (**A**) and NAA treatment (**B**). Width (**C**) and weight (**D**) of the peach fruit under mock and NAA treatment. Bars indicate the standard variation of the values (*n* = 30). Cross sections of mesocarp tissues derived from the fruit under 3-day mock (**E**) and NAA (**F**) treatments were shown in the representative 250-μm wide × 250-μm high regions; (**G**) cell densities were calculated by counting the number of the medium-sized cells regions in 250-μm wide × 250-μm high regions of cross section slides. Bars gives standard deviations (*n* = 15). NAA, α-naphthylacetic acid.

To explore the molecular regulation of auxin in peach fruit development, we treated the fruit of the peach cultivar Jing-Yan/Beijing24 (JY, *Prunus persica*, 2*n* = 16) at the hard core stage with 2 mM of the synthetic auxin NAA and mock solution (NAA omitted) and collected samples three days after treatment. NAA-treated fruits were significantly wider and heavier than those given mock treatment (*p*-value of *t*-test <0.01, *n* = 30; Figure 1A–D). It has been long known the auxin can regulate fruit development mainly through stimulating fruit cell enlargement; whereas cytokinin can enhance cell division [23,24,25]. We checked cell densities of mesocarp tissues derived from the fruit under mock (E) and NAA (F) treatments by cross section (Figure 1E–G). We observed the mesocarp cells were enlarged under NAA treatment compared with those following mock treatment. We subsequently isolated the mesocarp tissues from the fruit for small RNA profiling analysis.

**Figure 2:**
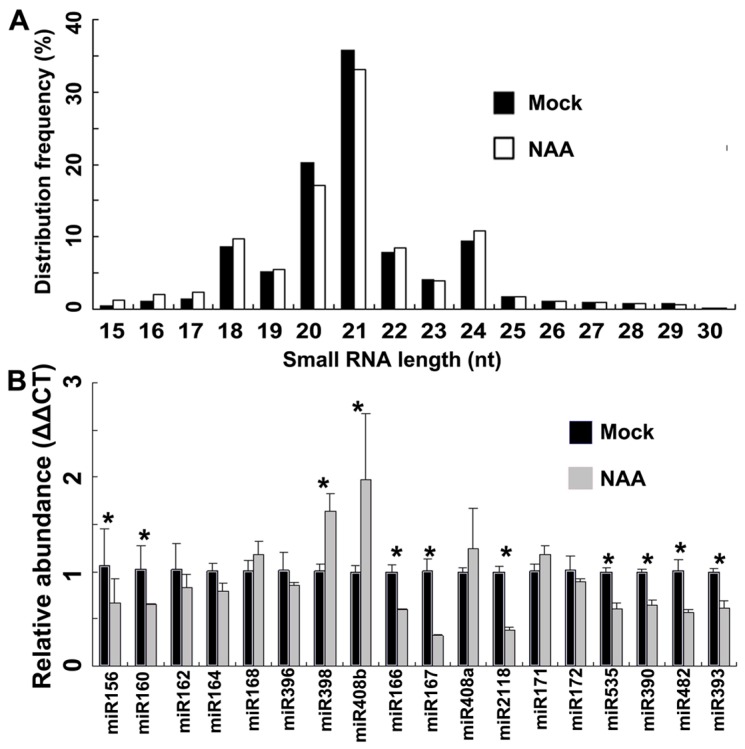
Identification of microRNA (miRNA in peaches by high throughput sequencing. (**A**) Length distribution of small RNA reads; (**B**) expression levels of 18 evolutionarily conserved miRNAs in mock- and NAA-treated samples using fluorescence stem-loop quantitative real-time PCR. * gives the significant difference (*p*-value <0.05). NAA, α-naphthylacetic acid.

Using our previously reported methods with minor modifications to preform small RNA (smRNA) sequencing experiments [26], we removed the adaptor sequences and aligned the sequenced RNAs with peach reference genome sequences [1]. A total of 726,827 and 1,263,079 mapped smRNAs and/or short RNA fragments were identified in the mock- and NAA-treated samples, respectively. The length distribution mainly ranged between 18-nt and 24-nt and around 33–35% were 21-nt smRNAs (Figure 2A). These results are consistent with a previous study [26].

We first searched our datasets for evolutionarily conserved peach miRNA genes. For this purpose, we compared our small RNA datasets with known miRNA sequences deposited in the miRbase and predicted their hairpin-structured precursors based on the flanking regions of the mapped genomic sequences using RNAfold [27]. We considered only those miRNA sequences with miRBase-annotated mature miRNAs and the predicted hairpin-structured precursors as evolutionarily conserved peach miRNAs. Our analyses identified 24 conserved peach miRNAs and we list their mature sequences and read count values derived from the control and NAA-treated mesocarp samples (Table S1).

Next, we parsed the genomic flanking regions of the 20–22 nt mapped smRNAs without any miRBase records and predicted their secondary structures. Our analyses revealed 1159 and 1722 smRNAs with predicted hairpin-structured precursors in mock- and NAA-treated samples, respectively. Subsequently, we referred the wildly used criteria to further identify miRNAs [28]. In brief, the miRNAs should have a RNA* derived from opposite stem-arms; the base-pairing between the miRNA and the other arm of the hairpin should have less than five mismatched bases; and asymmetric bulges should contain less than three bases. The high-quality miRNA candidates identified in treated and untreated samples were listed in Table S2. These high-quality miRNA genes with hairpin structures can be considered as newly identified miRNA genes or unannotated miRNA genes in peach.

miRNAs are generally accepted to act as post-transcriptional regulators, the functions of which show clear dosage effects. Accordingly, the highly and/or specifically expressed miRNAs are more likely to play versatile biological roles than those that have low abundance [29,30]. In our datasets, we detected twelve miRNA genes that were highly expressed (read counts >500). Of these, ten (83%) were conserved peach miRNAs, including miR160, miR162, miR164d, miR166, miR168, miR396a, miR396b, miR398, miR408a and miR482 (Table S1). The other two, miRC1 and miRC12a, were newly identified miRNAs (Table S2). miR160, miR162, miR164d, miR396b, miR408a were upregulated under NAA treatment, whereas miR166 was downregulated. These results suggest that the evolutionarily conserved miRNAs, especially those in high abundance, may play critical roles in regulating fruit development. Therefore, we mainly investigated the mRNA targets of these conserved miRNAs.

### 2.2. Experimental Verification of Important miRNAs in Peach

We further verified the expression levels of 18 evolutionarily conserved mature miRNAs in mock- and NAA-treated samples with three biological replicates using stem-loop qRT-PCR (Figure 2B). The abundance of 18s rRNAs were used as internal controls to normalize miRNA abundance and Fisher's exact test was performed to estimate the statistical significance of miRNA expression levels between mock and NAA-treated samples [31]. The expression levels of miR171, miR168, miR408a, miR398 and miR408b were significantly upregulated in mesocarp in NAA-treated samples compared to the control fruits, whereas those of miR156, miR160, miR166, miR167, miR390, miR393, miR482, miR535 and miR2118 were downregulated following NAA treatment. The experimental verification results were consistent with our high throughput sequencing datasets and also with previous studies that demonstrated the critical roles of miR160, miR166, miR167, miR390 and miR393 and their targeted genes in auxin signaling pathways. Our study further extends the functions of these miRNAs in fruit development.

### 2.3. Genome-Wide Identification of miRNA Targets by Degradome Sequencing in Peach

In plants, miRNA-directed cleavage of targeted mRNAs can temporarily produce mRNAs with 5’ uncapped ends, which can be captured and detected with a degradome sequencing platform [32]. To further explore the regulatory roles of the identified peach miRNAs on a genome-wide basis, we performed degradome sequencing experiments on matched control and NAA-treated mesocarp samples. Using alignment, we obtained 309,863 and 722,534 sequencing reads that uniquely matched to the reference mRNA sequences from the mock- and NAA-treated samples, respectively (Table S3).

In a degradome sequencing experiment, a true cleavage site of miRNA is expected to display a specific enrichment peak(s) of degradome reads on the mRNA that complementarily match the 8~10th nucleotides of the miRNA. To this end, we first predicted the putative cleavage sites in targeted mRNAs using psRNATarget [33] and then searched for read enrichment peaks around these putative cleavage sites. Finally, we categorized the prediction confidence levels of the miRNA cleavage sites into three levels according to their read enrichment on the targets: (1) high confidence level, wherein the cleavage site is located at the enrichment peak with the maximal read abundance on the targeted mRNAs; (2) intermediate confidence level, the cleavage site is located at the enrichment peak with read abundance ranging from average to maximal abundance on the targeted mRNAs; (3) low confidence level, a cleavage site is located at the enrichment peak with below average read abundances on targeted mRNAs. Our analyses identified 119 cleavage sites on predicted targets of the evolutionarily conserved miRNAs in peach following mock and NAA treatment. The genomic positions, confidence levels, normalized read counts, *p*-values and annotation of these miRNA-targeted genes are listed in Supplementary Table S4.

### 2.4. Regulation of Crucial Genes by miRNAs During Fruit Development

**Figure 3:**
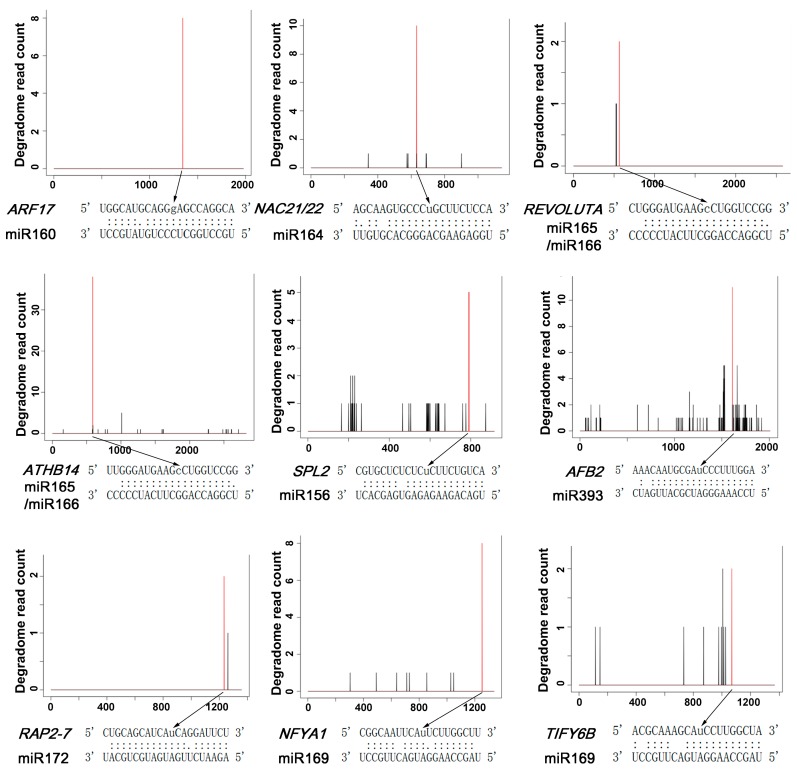
Identification of the cleavage sites of miRNAs by degradome sequencing in peaches. Cleavage sites of miRNAs on their targets were detected by degradome analysis. The *X*-axis indicates the mRNA position of miRNA targets from 5’ to 3’. The red bar shows the degradome read with the highest count on the mRNA. The miRNA duplex structures and their complementary regions on the targets are shown. Watson–Crick base pairs and G–U base pairs are indicated by “:” and “.”, respectively. Cleavage sites are indicated by black arrows and lower case nucleotides.

Genomics and genetics studies showed that miRNAs can affect several critical regulatory genes in auxin signaling pathways [14,15,34,35,36]. In our datasets, we also detected a subset of miRNAs and their cleavage sites (Table 1). We identified miR160 cleavage sites in *ARF17* mRNA with high prediction confidence in the peach fruit (Figure 3), which is consistent with previous studies in multiple plant species [14,15,34]. miR164 is known to target *NAC1* to downregulate auxin signal transduction pathway activity [37]. Our analysis also identified miRNA164 cleavage sites in mRNAs of the peach NAC family (Figure 3). Furthermore, we found that miR165/miR166 can target *REV*, *ATHB8*, *ATHB14* and *ATHB15* in the peach fruit with high prediction confidence (Figure 3). miR156 is known to target *SQUAMOSA* promoter binding protein–like (SPL) (Figure 3), but in our study, we found additional targets for miR156, including *ATHB13*, auxin-repressed protein (ARP), and inhibitor of growth protein 5 (ING5), suggesting that miR156 may have direct functions in modulating auxin response in the peach fruit. miR393 regulates expression of the auxin receptor gene TIR1 and the F-box genes *AFB1*, *AFB2* and *AFB3* [38,39]. Here we identified miRNA-directed cleavage events in *AFB2* (Figure 3). In addition to auxin signaling pathways, we also characterized the miR172 cleavage sites in the ethylene-responsive transcription factor gene *RAP2-7*, and sites for miR169 in nuclear transcription factor Y subunit A (*NFYA*) and *TIFY 6B* genes (Figure 3). These results suggest that a post-transcriptional regulation network of ethylene, abscisic acid (ABA) and/or jasmonic acid (JA) pathways is associated with fruit development and maturation. Taken together, our genome-wide analyses indicated that the evolutionarily conserved miRNAs and their targets in auxin signaling pathways may play crucial roles in regulating fruit development in peaches.

In addition, we compared our results with previously identified miRNA targets in the fleshy fruit of tomatoes [21]. We noted that miR156, miR172 and miR393 were also detected in tomatoes. miR156 and miR172 particularly targeted the SPL family gene colorless non-ripening (*CNR*) and the ethylene-responsive transcription factor gene *APETALA2a*, respectively, whereas miR393 targeted the AFB homolog gene *SlTIR1*. Therefore, our results were consistent with previous observations that miRNAs play evolutionarily conserved roles in fruit development and ripening by mediating the expression level of transcription factors (TFs).

## 3. Discussion

### 3.1. High-Throughput Sequencing Analysis

Using high-throughput sequencing and data analysis, we identified 726,827 and 1,263,079 small RNA sequences that matched to the genome in peach fruit following control and NAA treatment, respectively. Previous studies had profiled small RNAs in roots, leaves and other organs [7,8]. The amount of small RNAs in the peach fruit was relatively lower than that in roots and leaves. Nevertheless, we also identified a group of NAA-responsive miRNA genes. Most of these miRNA are evolutionarily conserved and has been defined in other species. Some of them are newly identified miRNA candidates that presented fruit-preferential expression pattern. Their biological functions highly warrant investigation. However, the functional validation of the novel miRNAs in peach fruit is time-consuming. The expression specificities, regulatory functions and evolutionary diversifications of these miRNAs need to be clarified in the future studies.

### 3.2. Identification of miRNAs in Peach Mesocarp

The results of real-time PCR experiments revealed the increased expression levels of miR171, miR168, miR408a, miR398 and miR408b, as well as the reduced expression levels of miR166, miR167, miR160, miR156, miR2118, miR535, miR390, miR482 and miR393 in the peach fruit after NAA treatment, respectively, suggesting the functional divergence of microRNAs in the regulation of fruit development. Among these miRNAs, miR160, miR166, miR167, miR390 and miR393 are known to play important roles in auxin signaling pathways [14,16,17,18,35,36].

### 3.3. miRNA Targets in Auxin Signaling Pathways

miR160 is known to target *ARF10*, *ARF16* and *ARF17* in multiple plant species [14,15,34]. Our results in the peach fruit were consistent with previous studies. We also identified miR165/miR166 cleavage sites in *ATHB8*, *ATHB14* and *ATHB15* in the peach fruit under control conditions. Moreover, TAS3 trans-acting short-interfering RNAs, which are targeted by miR390, can regulate miR166 expression that may control auxin flow via its target *HD-ZIP*. Together, these studies support an direct/indirect association between miR166 and auxin [35,36]. miR167 can target *ARF8*, *ARF6*, *TCP20* and *IAR3* to modulate plant growth and cell division [16,40,41,42]. In this study, we detected decreased expression levels of miR167 in the NAA-treated peach fruit. miR172 is known to play crucial roles in regulating fruit growth by mediating the expression of ARFs [19,20]. Hence, the slight reduction in miR172 expression seen in the NAA-treated fruit was in line with previous reports. miR390 was previously shown to repress the expression of ARFs by regulating tasiRNAs expression profiles [18]. In our study, miR390 expression levels were strongly reduced in the NAA-treated fruit, indicating that this miRNA may regulate fruit development by controlling expression of ARFs.

### 3.4. miRNA Targets in Other Pathways

In addition to auxin signaling pathways, we also characterized miRNA cleavage sites in mRNAs involved in ethylene, JA, ABA and other pathways. Our results showed that the expression levels of miR398 and miR408b increased after the 3-day NAA-treatment. miR398 was shown to cleave mRNAs encoding Cu/Zn superoxide dismutase to regulate oxidative stress responses [3,43,44,45,46]. miR169 can target *NFYA* to control dehydration responses [47]. Our experiment also identified a high confidence cleavage site for miR169 in peach *NFYA*. In addition, we detected miRNA cleavage sites on genes involved in stress responses. Some of these miRNA targeted genes can participate in plant hormone signal transduction processes by regulating the effect of biological and environmental stress factors. Based on the characterization of these putative targets of miRNAs, the biological functions of several of these genes should be investigated in future studies.

Furthermore, we found that some miRNAs were also identified in the fleshy fruit of the tomato, including miR156, miR172 and miR393 [21]. miR156 targeted the SPL family gene *CNR* that was reported to be involved in fruit ripening [48,49], and the overexpression of miR156 in tomatoes downregulated the weight and the number of fruit [50], miR172 targeted the ethylene-responsive TF *APETALA2a*, which negatively affects ethylene synthesis and positively affects fruit ripening. In the meantime, *APETALA2a* is positively regulated by *CNR*, indicating a regulatory feedback loop between miR172 and miR156 during fruit development and ripening [21]. miR393 targeted the AFB homolog gene *SlTIR1* that was shown to stimulate the fruit set and leaf morphogenesis in the tomato [51]. Taken together, these evidences elucidate that the evolutionarily conserved miRNAs play pivotal roles in fruit development and ripening via regulating the expression profiles of TFs.

## 4. Materials and Methods

### 4.1. Plant Materials and NAA Treatment

Peach trees of the Jing-Yan/Beijing 24 cultivar were grown in the field. We applied 2 mM NAA solution to the surface of the peach fruit (before harvest) at the hard core stage (seven weeks after full bloom), and used NAA omitted solution as the mock control, each treatment with three biological replicates. After three days, the fruit were harvested from trees and mesocarp tissues were isolated from the control and the NAA-treated fruit.

### 4.2. Sample Embedding

Along the ventral suture, the mesocarp of the peach fruit was cut into approximately 0.05 cm^3^ blocks. All excised samples were prefixed immediately in 2% (*w*/*v*) aqueous solution of 1-ethyl-3(3-dimethyl-aminopropyl)-carbodiimide hydrochloride (EDAC, Sigma, St. Louis, MO, USA) for 1h, then vacuumed and washed three times with 0.1 M phosphate-buffered saline (PBS, pH7.2). After washing, the samples were postfixed overnight in a solution containing 4% paraformaldehyde and 2.5% glutaraldehyde at 4 °C, dehydrated with *n*-butanol and ethanol, embedded in paraffin, and sectioned into 10-μm slices. The slides were spread with polylysine before the fixing of the sections. Dried sections were deparaffinized with xylene and hydrated in an ethanol-water series, then observed and photographed by Olympus BX51-400X microscope (Olympus, Japan). We selected 15 photos with clear view from the slides for counting cell numbers.

### 4.3. Small RNA and Degradome RNA Library Construction

Total RNAs were extracted using TRNzol-A+ Reagent (TIANGEN, Beijing, China) and RNase-free DNase treatment (Takara, Otsu, Japan). The concentrations were checked with a NanoDrop ND-1000 spectrophotometer. The RNA samples from three biological replicates were merged into one library for small RNA and degradome sequencing. Small RNA samples were extracted using a RNAmisi™ miRNA Isolation Kit (Aidlab, Beijing, China). RNA fragments ranging from 18 to 30 nt were gel-purified and ligated to 3′ and 5′ oligonucleotide adaptors. Reaction specificity was verified by melting curve analysis. We constructed a degradome library using a modified version of a previously described method [32]. In brief, about 150 ng poly (A)+ RNA was used as input RNA and annealed with biotinylated random primers. Only RNAs containing 5′-monophosphates can be captured and ligated with 5′ adaptors. After reverse transcription and PCR, the libraries were sequenced using the 5′ adaptor sequences.

The small RNA and degradome libraries were sequenced using an Illumina GAIIx sequencer (Illumina, San Diego, CA, USA) at the Beijing Genomics Institute (BGI), Shenzhen. The raw datasets of Small RNA and degradome sequencing experiments are available in the Sequence Read Archive of National Center for Biotechnology Information (NCBI) under accession SRP120910.

### 4.4. Bioinformatics Analysis for miRNA Identification

We used FastQC to check the sequencing quality of our datasets. Reads with low quality scores were filtered out. After trimming adaptors, the small RNA tags were queried with miRBase (Available online: http://www.mirbase.org/) to search for known miRNAs [52]. We aligned the small RNA tags to the peach reference genome using BWA with default parameters [53]. CleaveLand 3.0 was used to parse the alignment results [54]. The flanking regions of 19–22 nt small RNAs with more than three mapped sequencing reads were parsed according to their genomic positions. The secondary structures of pre-miRNAs were predicted using RNAfold software with the free energy parameter ΔG < −25.0 kcal/mol [27]. We predicted putative targets of the miRNAs using the psRNATarget server with default parameters [33]. We used a highly cited method to identify new miRNAs in peach [28].

The adaptor, pollution and low quality sequences of the degradome sequencing datasets were trimmed using Trimmomatic and CleaveLand 3.0 [54,55]. The remaining read sequences were aligned to the peach reference genome release v1.0 with general feature format (GFF)-formatted annotation files [1]. The read count density on each gene was subsequently calculated for the predicted miRNA-targeted genes. The *t-plot* figures of degradome reads were generated using CleaveLand 3.0 [54].

### 4.5. Stem-Loop RT-qPCR Analysis

We used a method based on previously described procedures to perform stem-loop RT-qPCR analysis to quantify miRNA expression [56,57]. The reactions were performed using a MiniOpticon Real-Time PCR Detection System (Bio-Rad, Hercules, CA, USA) with SYBR Premix Ex Taq (Takara). Primers based on 18s rRNAs were used as internal markers to normalize miRNA abundance. Expression levels of miRNAs were calculated using the comparative “ΔΔ*C*_T_” method with triplicates [58]. The stem-loop primers and qRT-PCR primers are listed in Table S5.

## 5. Conclusions

In this study, we profiled small RNA sequencing and the degradome of developing peach fruit at the hard core stage following exposure to NAA. Our experiments identified a subset of miRNAs that respond to NAA treatment. Most of these miRNAs are evolutionarily conserved. By miRNA target prediction analyses, we detected 119 miRNA cleavage sites on target mRNAs, including those for the *SPL*, *ARF*, *NAC*, *ATHB*, *REVOLUTA*, *TCP* and *AFB* family genes. We also verified the abundance of 18 miRNAs using stem-loop qRT-PCR assay. Our results indicated that the conserved miRNA and their targets in auxin signaling pathways played important roles in regulating fruit development at a post-transcriptional level in peaches.

## Figures and Tables

**Table 1:** List of conserved miRNAs obtained from control and α-naphthylacetic acid (NAA)-treated mesocarp of peaches.

miRNA ID	Target ID	Mock Cleavage Site	Mock Confidence	Mock *p*-Value	Mock Count	NAA Cleavage Site	NAA Confidence	NAA *p*-Value	NAA Count	Target Annotation
miR156	ppa012607m	791	High	8.00 × 10^-3^	5	N/A ^1^	N/A	N/A	N/A	Squamosa promoter-binding protein 2
miR156	ppa009498m	63	Intemediate	2.82 × 10^-2^	0.3	N/A	N/A	N/A	N/A	Homeobox-leucine zipper protein ATHB-13
miR156	ppa013510m	629	Intemediate	4.42 × 10^-2^	0.5	N/A	N/A	N/A	N/A	Auxin-repressed 12.5 kDa protein
miR156	ppa010764m	N/A	N/A	N/A	N/A	19	high	2.02 × 10^-2^	3	Inhibitor of growth protein 5
miR157	ppa012607m	791	High	5.54 × 10^-3^	5	N/A	N/A	N/A	N/A	Squamosa promoter-binding protein 2
miR160	ppa003136m	1346	High	2.09 × 10^-3^	8	1346	high	3.00 × 10^-3^	3	Auxin response factor 17
miR164	ppa008801m	632	High	1.36 × 10^-3^	8	632	high	1.83 × 10^-3^	10	NAC domain-containing protein 21/22
miR165/miR166	ppa001378m	565	High	3.70 × 10^-3^	2	N/A	N/A	N/A	N/A	Homeobox-leucine zipper protein REVOLUTA
miR165/miR166	ppa001386m	574	High	3.70 × 10 ^-3^	6.5	N/A	N/A	N/A	N/A	Homeobox-leucine zipper protein ATHB-8
miR165/miR166	ppa001343m	589	High	3.70 × 10^-3^	38	N/A	N/A	N/A	N/A	Homeobox-leucine zipper protein ATHB-14
miR165/miR166	ppa001405m	615	High	9.88 × 10^-3^	6.5	N/A	N/A	N/A	N/A	Homeobox-leucine zipper protein ATHB-15
miR168	ppa000547m	N/A	N/A	N/A	N/A	478	high	2.50 × 10^-3^	4	Protein argonaute 1B
miR169	ppa006634m	1252	High	1.73 × 10^-3^	8	N/A	N/A	N/A	N/A	Nuclear transcription factor Y subunit A-1
miR169	ppa012173m	N/A	N/A	N/A	N/A	709	high	1.83 × 10^-3^	2	Nuclear transcription factor Y subunit A-7
miR169	ppa008065m	N/A	N/A	N/A	N/A	1069	high	4.03 × 10^-3^	2	Protein TIFY 6B
miR172	ppa021782m	N/A	N/A	N/A	N/A	1234	high	1.83 × 10^-3^	2	Ethylene-responsive transcription factor RAP2-7
miR393	ppa003465m	1612	High	1.85 × 10^-3^	6	1612	high	2.50 × 10^-3^	11	Protein AUXIN SIGNALING F-BOX 2
miR394	ppa004699m	2100	Intemediate	1.47 × 10^-2^	2	N/A	N/A	N/A	N/A	F-box only protein 6
miR396	ppa021277m	452	High	1.28 × 10^-3^	2	452	high	2.46 × 10^-3^	2	AtGRF9 (GROWTH-REGULATING FACTOR 9)
miR396	ppa019623m	749	High	1.28 × 10^-2^	2	N/A	N/A	N/A	N/A	AtGRF1 (GROWTH-REGULATING FACTOR 1)
miR396	ppa019752m	N/A	N/A	N/A	N/A	716	high	2.30 × 10^-2^	3	AtGRF8 (GROWTH-REGULATING FACTOR 8)
miR405b-p3	ppa008493m	71	High	4.15 × 10^-2^	2	N/A	N/A	N/A	N/A	Myb-like protein J
miR482-p5	ppa000031m	N/A	N/A	N/A	N/A	4660	high	2.94 × 10^-2^	2	Chromodomain-helicase-DNA-binding protein 5

^1^ N/A, not applicable.

## Supplementary Materials

Supplementary materials can be found at http://www.mdpi.com/1422-0067/18/12/2599/s1.
